# Pharmacological MRI with Simultaneous Measurement of Cerebral Perfusion and Blood-Cerebrospinal Fluid Barrier Function using Interleaved Echo-Time Arterial Spin Labelling

**DOI:** 10.1016/j.neuroimage.2021.118270

**Published:** 2021-09

**Authors:** Charith Perera, Ian F. Harrison, Mark F. Lythgoe, David L. Thomas, Jack A. Wells

**Affiliations:** a UCL Centre for Advanced Biomedical Imaging, Division of Medicine, University College London, London, United Kingdom; b Neuroradiological Academic Unit, Department of Brain Repair and Rehabilitation, UCL Queen Square Institute of Neurology, London, United Kingdom; c Leonard Wolfson Experimental Neurology Centre, UCL Queen Square Institute of Neurology, London, United Kingdom; d Wellcome Centre for Human Neuroimaging, UCL Queen Square Institute of Neurology, University College London, London, United Kingdom

**Keywords:** Choroid plexus, Blood-cerebrospinal fluid barrier, Blood-brain barrier, Arterial spin labelling, Mri, Pharmacological mri, cerebrovascular reactivity, vasopressin, caffeine, CO_2_, ageing

## Abstract

Pharmacological MRI (phMRI) studies seek to capture changes in brain haemodynamics in response to a drug. This provides a methodological platform for the evaluation of novel therapeutics, and when applied to disease states, may provide diagnostic or mechanistic information pertaining to common brain disorders such as dementia. Changes to brain perfusion and blood-cerebrospinal fluid barrier (BCSFB) function can be probed, non-invasively, by arterial spin labelling (ASL) and blood-cerebrospinal fluid barrier arterial spin labelling (BCSFB-ASL) MRI respectively. Here, we introduce a method for simultaneous recording of pharmacological perturbation of brain perfusion and BCSFB function using interleaved echo-time ASL, applied to the anesthetized mouse brain. Using this approach, we capture an exclusive decrease in BCSFB-mediated delivery of arterial blood water to ventricular CSF, following anti-diuretic hormone, vasopressin, administration. The commonly used vasodilatory agent, CO_2_, induced similar increases (~21%) in both cortical perfusion and the BCSFB-ASL signal. Furthermore, we present evidence that caffeine administration triggers a marked decrease in BCSFB-mediated labelled water delivery (41%), with no significant changes in cortical perfusion. Finally, we demonstrate a marked decrease in the functional response of the BCSFB to, vasopressin, in the aged vs adult brain. Together these data, the first of such kind, highlight the value of this translational approach to capture simultaneous and differential pharmacological modulation of vessel tone at the blood brain barrier and BCSFB and how this relationship may be modified in the ageing brain.

## 1 Introduction

The blood-brain-barrier (BBB) and blood-cerebrospinal fluid barrier (BCSFB) mediate the complex interplay between blood and the brain, which is a core homoeostatic mechanism supporting healthy brain function. The main locus of the BCSFB is the choroid plexus, which resides in the brain's fluid filled ventricles. We have recently developed a MRI technique for the non-invasive assessment of BCSFB function, by quantifying the rate of BCSFB-mediated delivery of endogenous arterial blood water to ventricular cerebrospinal fluid (CSF) (Evans et al., 2020). As such, this translational approach (termed blood-cerebrospinal fluid barrier arterial spin labelling [BCSFB-ASL]) may be useful to better understand the precise role of the BCSFB in conditions such as Alzheimer's disease and multiple sclerosis, where dysfunction has been postulated to be mechanistically significant (Balusu et al., 2016; Vercellino et al., 2008). Importantly, this technique allows repeated measures with high temporal resolution, which non-invasively enables dynamic capture of the BCSFB's functional response to drugs, challenges, and disease.

Estimates of cerebral blood flow (CBF) provide a surrogate measure of the functionality of the BBB to, for example, facilitate a constant supply of oxygen and nutrients from the blood to the highly metabolically active brain cells. CBF can be measured non-invasively with standard arterial spin labelling (ASL) MRI (Haller et al., 2016). ASL also provides repeated measures and so can be used to measure dynamic changes in CBF in response to a drug (an approach which falls under a set of methods known as pharmacological MRI [phMRI] (Zhou et al., 2015; Inoue et al., 2014)). phMRI provides a non-invasive means of assessing the spatial-temporal dynamics of new and emerging drugs to alter brain function and/or the brain's vascular properties. Such measurements can also provide novel diagnostic and/or mechanistic insight into brain pathology by examining how underlying disease states modulate the brain's vascular response to a drug (Kastrup et al., 1998; Suri et al., 2015; Hurford et al., 2014).

Due to the relatively long half-life of most drugs compared to the duration of an MRI scan, it is typically only possible to measure the functional response to a single dose in a given phMRI session. This limits the scope of MRI scan-types applied to characterize pharmacological perturbation of brain hemodynamics in real time. Indeed, to date, typically either ASL or T2* weighted blood-oxygenation-level-dependant (BOLD) measurements have been recorded in phMRI studies.

Here, we exploit the overlap of the traditional ASL and BCSFB-ASL MRI techniques and employ an interleaved echo time (TE) ASL sequence to capture the simultaneous response of two distinct components of brain physiology to a single dose of a drug or ‘challenge’: i) parenchymal tissue perfusion, and ii) rates of BCSFB-mediated arterial blood water delivery to the CSF (a surrogate, non-invasive, measure of BCSFB function). This approach provides an efficient means to better understand the differential response of vessels that comprise the BBB and BCSFB to pharmacological perturbation in the healthy and diseased brain. We first applied this method to reproduce the well-established specific vasoconstriction of vessels that perfuse the choroid plexus (with no decrease in parenchymal perfusion) that is induced by antidiuretic hormone, vasopressin (Evans et al., 2020; Faraci et al., 1988). Then, we investigated whether the commonly used vasodilatory agent, CO_2_, is a viable approach to dilate the vasculature of the BCSFB. Finally, given experimental data linking caffeine consumption to marked changes in choroid plexus physiology, we proceeded to measure the effect of caffeine on non-invasive measures of BCSFB function in the mouse brain.

There is wide-ranging evidence that marked deterioration of BCSFB structure and function occurs in the aged brain, with increased levels of endogenous vasopressin hypothesized to be an important mechanism underlying this decline (Frolkis et al., 2000; Liszczak et al., 1986; Dohrmann, 1970; Johanson et al., 1999; Sturrock, 1979). Therefore, to investigate the mechanistic link between BCSFB function and vasopressin in the aged brain, using non-invasive measurements, we applied the interleaved-TE ASL technique to capture the phMRI response to vasopressin in a cohort of aged and adult mice. Together, our findings represent the first phMRI measurements of BCSFB function and, based on the marked differential response to vasopressin in the aged brain, highlight the potential of this approach to better understand the mechanisms that underlie age related cognitive decline.

## 2 Methods

### 2.1 Animal preparation

All animal procedures were performed under the UK Home Office Act (Scientific Procedures, 1986). C57/BL6 female WT mice (provided by Charles River Laboratories) were used for the pharmacological experiments conducted only in adult mice. When investigating the effects of ageing on the brain, 14 aged mice (C57BL/6JRj 23-months old, male) and 14 strain-matched adult-mice (5-months old, male) were used, provided by Janvier Labs (France). Further details of sample sizes, dosing and administration routes for the pharmacological/gas challenge experiments are outlined in Table 1.

**Table 1:** Pharmacological/gas challenge administration. Details of the mouse strain and sample sizes used for our pharmacological/gas challenge experiments, alongside the doses and administration routes for each of the selected challenges, as well as the anaesthetic regimes. Timings of the experimental phases have been shown: baseline, challenge (“on” time), and recovery (for the hypercapnia challenge only).

Mouse strain/cohort	Sample size (n)	Pharmacological/Gas challenge	Dose	Administration route	Baseline (mins)	Challenge (mins)	Recovery (mins)
C57BL/6 Adult	9	Saline (vehicle)	5 ml/kg	Intraperitoneal bolus	10	20	–
C57BL/6 Adult	6	Vasopressin	47 U/kg	Intraperitoneal bolus	10	20	–
C57BL/6 Adult	6	Caffeine	50 mg/kg	Intraperitoneal bolus	10	20	–
C57BL/6 Adult	8	CO_2_	10%	Nose cone inhalation	10	10	10
C57BL/6JRj Adult (5-months)	14	Vasopressin	47 U/kg	Intraperitoneal bolus	10	20	–
C57BL/6JRj Aged (23-months)	14	Vasopressin	47 U/kg	Intraperitoneal bolus	10	20	–

Prior to commencing MRI acquisitions, subjects underwent anaesthetic induction using 2% isoflurane in 0.8 L/min medical air and 0.2 L/min O_2_. Following induction and weighing, mice were placed into the MRI cradle with bite bar, nose cone and ear bars to ensure a well secured position of the mouse head to minimise motion during the data acquisition. Eye ointment was also applied to prevent drying.

For subjects receiving vasopressin, caffeine, or saline vehicles, an intraperitoneal infusion line was attached to each subject to allow for the delivery of drug solution boluses whilst the animal was positioned within the magnet bore. The length of the line allowed manual injection from outside the scanner bore, without needing to reposition the cradle. A scavenger pump was placed inside the magnet bore to prevent isoflurane build-up. Anaesthesia was maintained during the acquisition by reducing isoflurane concentration to 1.5% in 0.4 L/min medical air and 0.1 L/min O_2_.

For the CO_2_ experiments, the animals were induced using and initially maintained using isoflurane (as described above). Once in the cradle, a separate infusion line was attached subcutaneously to allow for the delivery of a 0.4 mg/kg bolus of medetomidine (5% in saline) via a Syringe Pump (Infuse/Withdraw PHD 22/2000, Harvard Apparatus). Following this, isoflurane concentration was reduced to 0.5% with 1.0 L/min medical air, well before commencing dynamic MRI data capture. During the acquisition, anaesthesia was additionally maintained by subcutaneously infusing the medetomidine at a rate of 0.8 mg/kg/hour.

Temperature and breathing rate were monitored throughout all the experiments using a rectal probe and a respiration pad (SA Instruments). Mouse temperature was maintained at 37 ± 0.5 °C using a combination of heated water tubing and warm air flow during both the induction and data acquisition stages.

### 2.2 Pharmacological challenge administration

For the hypercapnia protocol, after obtaining a 10-minute baseline, medical air was substituted for CO_2_ (10%) delivered at 1 L/min in medical air for 10 min. Subsequently, CO_2_ was replaced again by medical air for another 10 min to induce a recovery period. In the case of vasopressin, caffeine, and saline vehicles, after a 10-minute baseline, the drug solution was manually injected, and measurements were obtained for 20 min.

### 2.3 Magnetic resonance imaging (MRI) protocols

Images were acquired using an Agilent 9.4T imaging system with a 72 mm volume coil for RF transmission and a two-channel array surface coil receiver (Rapid Biomedical), positioned on top of the head.

### 2.4 Anatomical reference scans

Anatomical reference structural images were acquired with T2 weighting in order to clearly visualise the location of the major CSF compartments in the mouse brain using a fast-spin echo sequence (FOV = 25 mm x 25 mm; matrix size = 256 × 256; echo train length = 8; TEeff = 48 ms; TR = 5 s).

Sagittal anatomical reference images (8 slices, 1 mm slice thickness) were used to position the axial anatomical reference imaging slice and the ASL imaging slices (Supplementary Figure 1a).

Coronal anatomical reference images (6 × 0.4 mm slices, 2.4 mm total) were manually positioned to align with the caudal region of the lateral ventricles to display the corresponding imaging volume across the ventricles (Supplementary Figure 1d).

### 2.5 FAIR-ASL scans

Each of the ASL imaging protocols was based on the FAIR sequence (Kim, 1995) with a single shot SE-EPI readout, slice-selective width = 19.2 mm, and a global labelling pulse, across all the experiments.

Parameters for standard-ASL and BCSFB-ASL: single slice, 2.4 mm slice thickness, TR = 12,000 ms, TI = 4000 ms, matrix size: 32 × 32, FOV: 20 mm x 20 mm, inversion pulse BW = 20,000 Hz, shape = sech. 38 repetitions were used to cover a 30-minute scan protocol. TE = 20 ms for standard-ASL, and TE = 220 ms for BCSFB-ASL.

Importantly, the ASL imaging slice was manually positioned to align with the caudal end of the lateral ventricles, as it has been previously shown to be the predominant region within the lateral ventricles which the CP occupies (Lein et al., 2007) (Supplementary Figure 1a). Therefore, as described in our recent work (Evans et al.) (Evans et al., 2020), our measurements of BCSFB function are concentrated to CP within the later ventricles and not the 3rd and 4th ventricle.

**Fig. 1:**
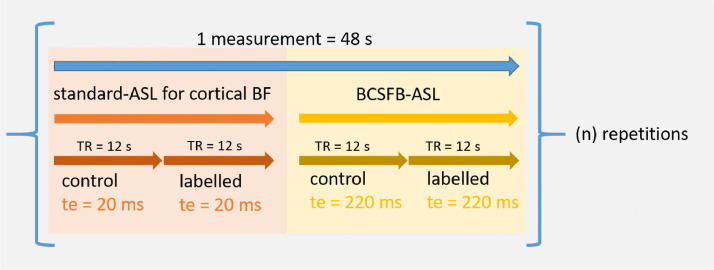
Interleaved echo time ASL acquisition. Within a single 48 second repetition, 2 pairs of controlled and labelled images are obtained at echo times of 20 ms and 220 ms.

In order to interleave a traditional-ASL measurement with the BCSFB-ASL measurement, an additional array loop was added to switch between two echo time values: 220 ms (BCSFB-ASL) and 20 ms (traditional-ASL). As shown in Fig. 1 in a single repetition of the interleaved-ASL technique (48 s), there are 4 ASL images obtained, each with a TR of 12 s: standard-ASL control image (20 ms), standard-ASL labelled image (20 ms), BCSFB-ASL control image (220 ms), BCSFB-ASL labelled image (220 ms). This loop continues for a given number of repetitions.

### 2.6 Image processing and analysis for relative blood flow quantification

When analysing standard-ASL images obtained with TE = 20 ms, a single ROI was drawn for each subject across the cortex of the brain using the slice-selective FAIR image, and the mean voxel signal was calculated across the ROI. For each ASL image pair, the non-selective mean ROI value was subtracted from the slice-selective mean ROI value to provide the perfusion-weighted signal ΔM, which was then divided by the corresponding control magnetisation (non-selective signal, M_c_).

For the BCSFB-ASL images obtained with TE = 220 ms, two 3 × 2 voxel ROIs (12 voxels in total) were positioned on a slice-selective image, overlaid with the position of the lateral ventricles (Supplementary Figure 1c). As with the standard-ASL analysis, the combined ROI average signals were subtracted in a pairwise fashion to provide ΔM values.

For both standard- and BCSFB-ASL data, in a subject-wise manner, ΔM/M_c_ values were divided by the mean ΔM/M_c_ value for the 10-minute baseline to provide a measure of relative, baseline-normalised blood flow. The average normalised ASL signal during baseline was taken and compared to the average normalised signal following the administration of vasopressin/caffeine or during the period of CO_2_ administration, using a paired *t*-test. A ten-minute analytical window at the end of the challenge period was used for IP challenges (vasopressin, caffeine, saline). The full ten-minute window was also averaged for the hypercapnia challenge period. At this stage of technique validation, this summary measure provides a straightforward and conservative quantification of the degree of response towards pharmacological or gas challenges.

### 2.7 Mono-exponential model of ASL response to pharmacological challenges

The mean time-course data for vasopressin and caffeine were fitted to a simple mono-exponential decay model for the purpose of providing further visualisation of responses towards pharmacological challenges. A piecewise function was used, with the following equation:

$$y\;=\{\;\begin{array}{c} b,\;x<10\\ b.exp\left(d.\left(x-10\right)\right),\;x\geq 10 \end{array}$$

where *x* = time (mins), *y* = relative blood flow, *b* = baseline blood flow, *d* = mono-exponential decay constant. The switching point, *x* = 10 mins, corresponds to the point when challenges were administered following the baseline.

## 3 Results

**Fig. 2:**
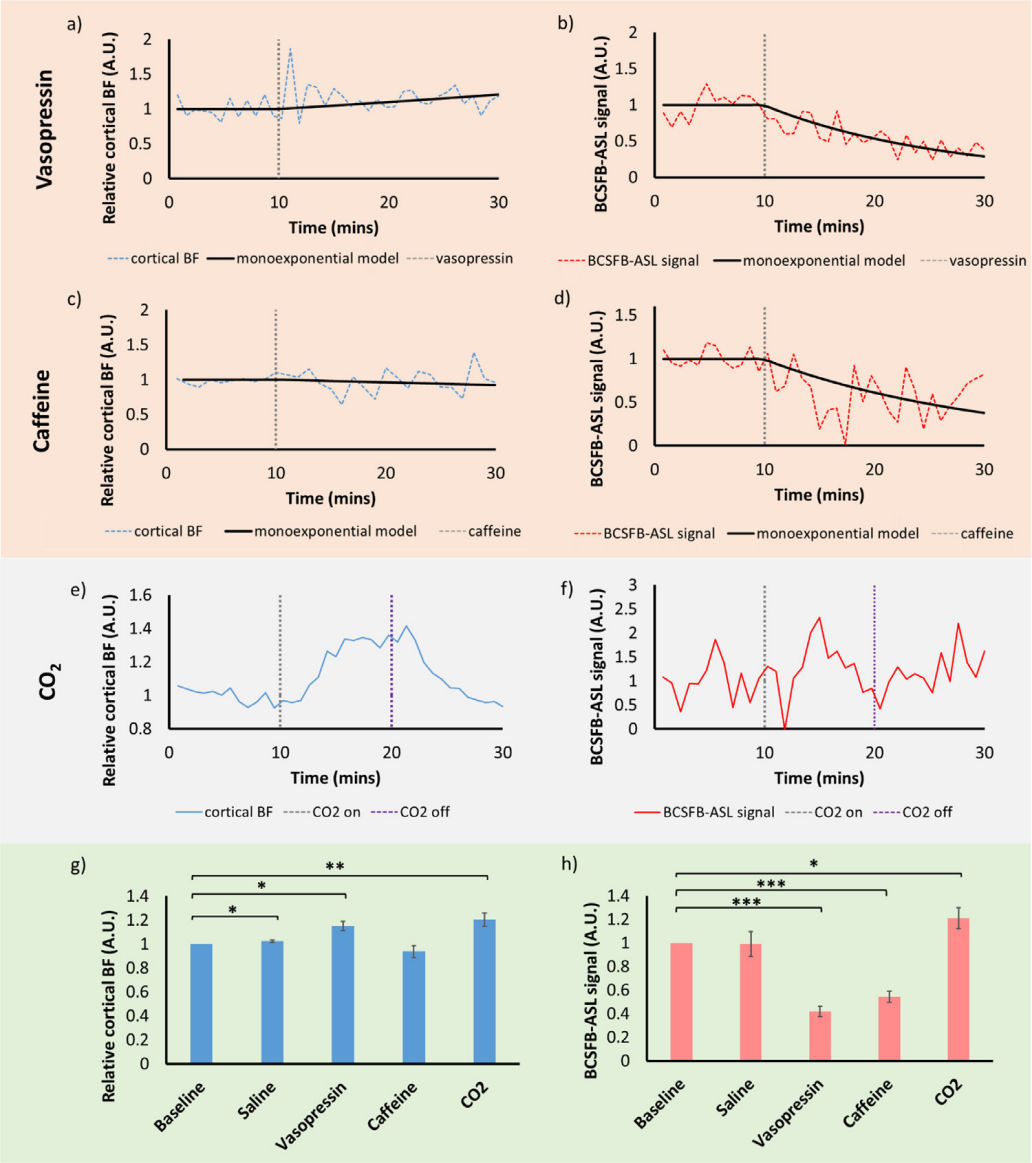
Interleaved echo time ASL: cortical BF and BCSFB-ASL simultaneous responses to selected pharmacological/gas challenges. Top row: vasopressin. Second row: caffeine. Third row: CO_2_ (following a distinct experimental routine compared to the IP challenges). Averaged time courses alongside the mono-exponential model fittings are shown for cortical BF (a, c, e,) and the BCSFB-ASL signal (b, d, f). Bottom row: bar plots displaying group-averaged relative changes from baseline during a window in the challenge period, for cortical BF (g) and the BCSFB-ASL signal (h). Error bars: ±SEM.

Interleaved-TE ASL measurements were obtained to investigate the effects of vasopressin, CO_2_, and caffeine on cortical perfusion and rates of BCSFB-mediated arterial blood water delivery to ventricular CSF in the C57BL/6 WT adult mouse brain (*n* = 6). Administration of saline vehicle revealed very subtle changes in cortical perfusion, and no measurable change in the BCSFB-ASL signal (ASL signal: 2.3% mean change from baseline, *p* = 0.03; BCSFB-ASL signal: −0.9% mean change from baseline, *p* = 0.92; *n* = 9, Fig. 2g, h).

### 3.1 Vasopressin

Evidence from previous invasive measures, as well as previous non-interleaved, BCSFB-ASL acquisitions in our lab, has highlighted the ability of vasopressin to cause specific vasoconstriction at the BCSFB (with no decrease in cortical perfusion) (Evans et al., 2020; Faraci et al., 1988). Fig. 2 (a, b) shows the simultaneous response of cortical perfusion and the BCSFB-ASL signal to a vasopressin challenge in 6 mice, using the interleaved echo-time ASL phMRI protocol. Averaged dynamic cortical blood flow time course data revealed a significant, 15% average increase from baseline upon vasopressin administration (*p* = 0.010, Fig. 2g). Conversely, the BCSFB-ASL time course data revealed a significant vasopressin-induced decrease (mean change 58%, *p* = 0.00004, Fig. 2h). In summary, as hypothesised, the application of a pharmacological vasopressin challenge evoked significant and specific downregulation of BCSFB function from baseline values, with no evidence for a decrease in cortical perfusion.

### 3.2 Caffeine

Caffeine is a safe and commonly consumed drug (van Dam et al., 2020) which has been repeatedly implicated as a significant modulator of BCSFB physiology (Han et al., 2009). Here we explored the potential of caffeine to pharmacologically challenge the BCSFB, with functional changes captured using non-invasive methods for the first time. Fig. 2 (c, d) shows the simultaneous response of cortical blood flow and rates of BCSFB-mediated blood water delivery to the CSF in response to a caffeine challenge. Imposing a caffeine challenge revealed significant decreases in the BCSFB-ASL signal by an average of 46% (*p* = 0.0002, Fig. 2d, h). However, caffeine administration did not evoke significant changes in cortical perfusion (*p* = 0.27, Fig. 2g).

### 3.3 CO_2_

CO_2_ is a vasodilatory agent commonly used to challenge the brain's vasculature, with the extent of the haemodynamic ‘reactivity’ often interpreted as a measure of cerebrovascular health (Zhou et al., 2015; Kastrup et al., 1998; Suri et al., 2015; Hurford et al., 2014). Here we were interested in investigating possible CO_2_-driven changes to vessel tone in the choroid plexus using MRI for the first time. Fig. 2 (e, f) shows the simultaneous response of cortical blood flow and the BCSFB-ASL signal in response to a hypercapnia challenge. As expected, following the administration of a hypercapnia challenge, there was a significant increase in cortical perfusion (*p* = 0.0038), averaging 21% (Fig. 2g). Furthermore, significant increases in the BCSFB-ASL signal from baseline were also detected upon inducing hypercapnia averaging 21% (*p* = 0.031, Fig. 2h).

Previous studies have reported elevated levels of endogenous vasopressin with ageing (Frolkis et al., 2000; Keck et al., 2000; Johnson et al., 1994). As vasopressin is associated with reduced CP perfusion (see, for example, Fig. 2d), this is thought to contribute to the established impairment of CP structure and function in the aged brain (Johanson et al., 1999).

To further probe the mechanistic interaction between BCSFB function, vasopressin and brain ageing using non-invasive methods, we applied the interleaved-TE ASL technique to a cohort of adult (5-months, *n* = 14) and aged (23-months, *n* = 14) mice to measure the response of the vessels within the BCSFB to a vasopressin challenge. We hypothesised a reduced response to exogenous vasopressin in the aged cohort, owing to the established elevation of endogenous levels of vasopressin in blood plasma with ageing (Frolkis et al., 2000).

**Fig. 3:**
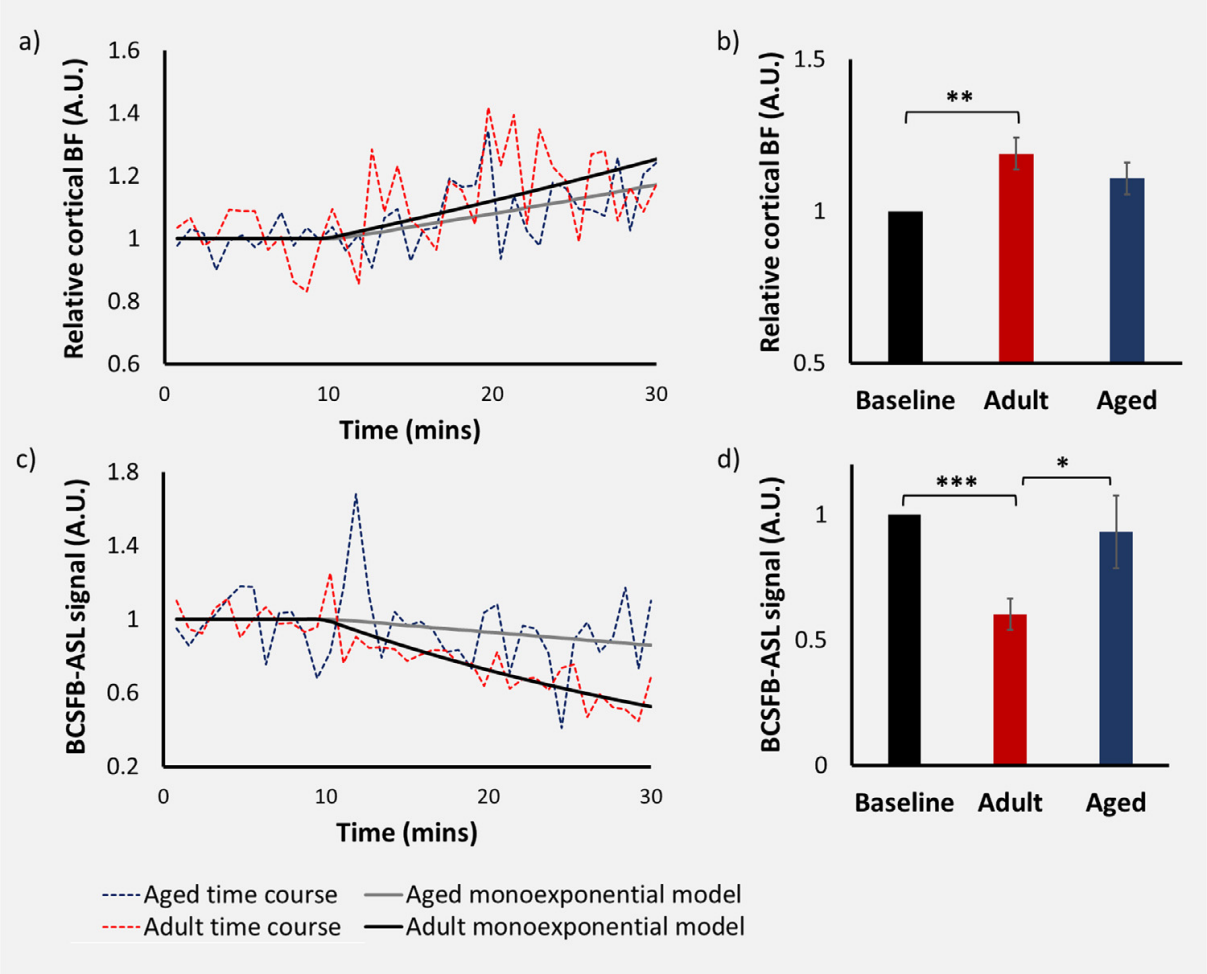
Interleaved echo time ASL: adult vs aged response to vasopressin. Top row: cortical BF response to vasopressin. Bottom row: BCSFB-ASL signal response to vasopressin. Averaged time courses alongside the mono-exponential model fittings are shown for both adult and aged cohorts, for cortical-BF (a) and the BCSFB-ASL signal (c). Bar plots display averaged relative changes in the adult and aged groups relative to baseline, for cortical BF (b) and the BCSFB-ASL signal (d). Error bars: ±SEM.

Fig. 3 shows the cortical BF and BCSFB-ASL signal responses following vasopressin administration, in 14 adult and 14 aged mice, using an interleaved-TE ASL approach. We observe significant increases in cortical blood flow following vasopressin administration in adult mice averaging 19% (*p* = 0.003, data Fig. 3a, b). Increases in average cortical perfusion in aged mice bordered significance (11% average *p* = 0.057, Fig. 3a, b). Post-hoc analysis revealed that there was no significant difference between the cortical reactivity to a vasopressin challenge between the adult and aged cohort (*p* = 0.28).

As expected, vasopressin induced a marked decrease in the BCSFB-ASL signal in the adult cohort, averaging 40% (*p* = 0.00002, Fig. 3c, d). In contrast, the response to vasopressin in the aged cohort was dampened, averaging a 6.8% decrease (not significant, *p* = 0.64, Fig. 3c, d). The marked 6-fold impairment in the response of the BCSFB-ASL signal observed in aged mice vs their adult counterparts was statistically significant (*p* = 0.046, Fig. 3d).

## 4 Discussion

To date, studies investigating BCSFB function *in-vivo* have been limited in number. Historically, obtaining measurements of choroid plexus perfusion necessitated the use of highly invasive techniques with injectable radiotracers or contrast agents alongside terminal surgical procedures, and hence were limited in their temporal resolution and translatability (Hubert et al., 2019; Prinzen and Bassingthwaighte, 2000). The BCSFB-ASL technique provides a surrogate measure of BCSFB function by quantifying the rate of BCSFB-mediated delivery of endogenous arterial blood water to ventricular CSF, by using an ultra-long TE (220 ms @ 9.4T), low spatial resolution readout ASL sequence. Here, by arraying the ultra-long and short TEs used for BCSFB- and traditional-ASL, respectively, and keeping all other parameters constant, the interleaved-TE ASL approach provides a platform to non-invasively monitor simultaneous, dynamic changes in brain parenchymal perfusion and BCSFB-function in the mouse brain.

Here, we demonstrate the application of this sequence to the field of phMRI, where it may be particularly advantageous, since typically only a single dose of drug can be administered in a single imaging session. We demonstrate differential responses to vasopressin and caffeine, highlighting the distinct physiology of the BBB associated with vessels that perfuse the cortex and the BCSFB within the CP. Moreover, we apply the method to probe the mechanistic interaction between BCSFB function, ageing, and vasopressin, recording a dampened pharmacological response at the BCSFB to exogenous vasopressin in the aged mouse brain.

The BCSFB-ASL signal reflects the average rate of perfusion to the CP convolved with the permeability of the BCSFB to water (i.e. the ‘extraction fraction’ (Raichle et al., 1974)) and the mass of the CP tissue (in this case, within the lateral ventricles). It is important to note, therefore, that this measurement reflects the rate of delivery of labelled blood water across the BCSFB rather than the net secretion of CSF. We speculate that changes to the BCSFB-ASL signal driven by pharmacological/gas challenges measured here are primarily driven by alteration in vessel tone at the BCSFB, in turn modulating CP perfusion. For example, vasopressin is known to cause vasoconstriction at the CP (Faraci et al., 1988) but increase vessel permeability to water (Raichle and Grubb, 1978); we measure a decreased BCSFB-ASL signal, suggesting that vasoconstriction is the dominant mechanism. Indeed, vasopressin evoked a marked (58%) decrease in BCSFB-mediated blood water delivery to the CSF. Reproducing this finding, previously reported both with an invasive microsphere approach (Faraci et al., 1988) and with un-interleaved BCSFB-ASL measurements (Evans et al., 2020), demonstrates the sensitivity of interleaved-TE ASL to detect an exclusive downregulation in BCSFB function, as hypothesised.

Hypercapnia-induced cerebrovascular reactivity (CVR) measurements using MRI-based approaches are frequently conducted both pre-clinically and clinically. Dampened dilatory responses to CO_2_ have been implicated in many pathological conditions affecting the brain microvasculature, such as cognitive decline in ageing and dementia, hypertension, as well as being associated with a higher risk of ischaemic injuries (Zhou et al., 2015; Kastrup et al., 1998; Suri et al., 2015; Hurford et al., 2014). We report significant increases in cortical perfusion under hypercapnia in the mouse brain, as hypothesised, alongside simultaneous increases in the BCSFB-ASL signal to a similar magnitude. Despite the established increases in CBF following CO_2_ administration, there is limited and conflicting literature detailing the effects of hypercapnia to CP perfusion. Hypercapnia has been shown to significantly increase CP blood flow in sheep by 27% (Page et al., 1980), with other studies contradicting this finding by reporting an approximately 2-fold decrease in response to CO_2_ (Nakamura and Hochwald, 1983). Autoradiography measurements in rats did not capture any CO_2_-driven changes in CP blood flow (Williams et al., 1991). Our results indicate a significant, CO_2_-driven increase in CP perfusion of a similar magnitude to that observed in the cortex.

Measurements of caffeine-induced CBF changes in humans and rat models have shown global and regional CBF decreases (Addicott et al., 2009; Vidyasagar et al., 2013; Nehlig et al., 1990). However, there are currently no available reports detailing the effects of caffeine on CBF in the mouse brain specifically. We provide dynamic data covering the immediate effects of caffeine, with no significant decreases in cortical BF observed here. The lack of significant decrease in cortical BF observed here may reflect a type II error, as prior studies would suggest any putative decrease to be relatively subtle. Nonetheless, our data clearly demonstrates the magnitude of caffeine-driven decreases in the BCSBF-ASL signal to be markedly greater than changes in cortical perfusion, shown to be significant through post-hoc testing, *p* = 0.0006). Caffeine has been found to be a potent modulator of BCSFB physiology (Han et al., 2009). Indeed, the marked decrease of the BCSFB-ASL signal suggests that the vasculature of the BCSFB is more dramatically affected by caffeine-induced vasoconstriction than the vessels in the cortex. The dose applied here, when allometrically scaled for human administration, equates to approximately 2 espresso coffee shots, thus keeping translatability as an important consideration (Nair and Jacob, 2016). By virtue of the convenient and commonplace ingestion of caffeine outside the realms of blood flow studies in humans, caffeine becomes an ideal candidate for studying the functional response of the BCSFB in a future clinical setting.

Ageing remains the primary risk factor for the development of dementias such as Alzheimer's Disease, as well as other neurodegenerative conditions such as Parkinson's disease (Van Der Flier and Scheltens, 2005; Hou et al., 2019). The increased prevalence of these age-associated disorders within our sociecty calls for a deeper understanding of the changes which renders an ageing brain vulnerable to these pathologies. It has become increasingly evident that the CP-BCSFB locus undergoes numerous morphological and functional changes within the ageing brain, with associated impairment of the BCSFB's role in secretory, transport, immune, and barrier function (Balusu et al., 2016; Vandenbroucke, 2016; Serot et al., 2001). These changes include hypoperfusion of the CP (Evans et al., 2020), impaired CSF production and turnover (Redzic et al., 2005), changes in epithelial cell metabolism and transport (Chen et al., 2009), the deposition of various species within the cell cytoplasm of the epithelial cells, and calcification of the basement membrane (Marques et al., 2013). Obtaining novel measurements of BCSFB reactivity may provide a source for upstream disease biomarkers which give insights into the barrier's functionality and improve the current understanding of early pathological events.

Significant increases in endogenous levels of vasopressin in the blood have been reported in ageing, both in humans and in animal models (Frolkis et al., 2000; Keck et al., 2000; Johnson et al., 1994). At the BCSFB, vasopressin binds to V1 receptors situated on the CP epithelial cell (CPec) membrane (Faraci et al., 1988). Alongside the subsequent vasoconstriction to decrease CP blood flow and decrease CSF secretion, this binding event can induce a transition towards the formation of dark CP epithelial cells (Sturrock, 1979), observed distinctly *in-vitro* and *in-vivo*. Although the study of these dark cells has been limited, they have been shown to occur naturally *in-vivo*, in the adult mammalian brain of mice, dogs and humans (Liszczak et al., 1986; Dohrmann, 1970). Previous work comparing infant to adult rats have shown increases in the number of dark cells within the BCSFB with age, linked to the aforementioned increase in endogenous vasopressin plasma concentrations with ageing. A more prominent dark cell population modifies hemodynamics and fluid transfer across the BCSFB, i.e. decreasing both perfusion to the CP and CSF secretion rates (Johanson et al., 1999). Therefore, it is possible that the increases in endogenous vasopressin, associated with increased dark cell formation, may contribute to decreases in the reactivity, perfusion, and secretory efficiency of the BCSFB with age. We hypothesised that as a result of the impairment in functionality following the changes in structure and function of the BCSFB, combined with the increased level of endogenous vasopressin within the aged brain, the aged mouse brain will display a decreased magnitude of reactivity towards a vasopressin challenge relative to their strain-matched adult counterparts. Aged mice were shown to have a significant, dramatic reduction, in their reactivity to a vasopressin challenge when compared to adult mice. As such, this finding represents the first demonstration of a differential response of the BCSFB to a pharmacological challenge across different brain states (in this case the aged vs adult mouse brain). It would be interesting to apply the methods to mouse models of neurodegenerative conditions such as AD in future studies.

In this work, blood pressure was not recorded. However, this is unlikely to be a significant confound for data interpretation as: i) in the case of caffeine, several studies have shown that at clinically relevant doses, caffeine causes a relatively subtle increase in BP that is well within autoregulatory limits (Flueck et al., 2016); ii) for CO_2_, previous studies have shown that increased CBF is primarily driven by local vasodilation in the brain and not systemic changes in BP (Kety and Schmidt, 1948; Wagerle and Mishra, 1988); iii) vasopressin is known to increase BP, however we observe a significant decrease in the BCSFB-ASL signal, suggesting that local changes in vessel tone are the dominant mechanism underlying this change.

Currently, the intraperitoneal dose of vasopressin used in our experiments equates to approximately 45 U/kg. This dose, was chosen to provide a robust and reproducible response to maximise sensitivity in the mechanistic study on the ageing mice, based on previous measurement in our lab (Evans et al., 2020). Allometric conversion to a human dose gives an approximate dose of 4 U/kg. Vasopressin is used clinically, albeit not for vascular reactivity protocols, at a dosing range 1–2 orders of magnitude lower than our chosen dose (Vasostrict, 2021; Mitra et al., 2011). Thus, there is potential for refinement in the dosing, delivery, and/or imaging paradigm to enable the measurement of BCSFB functionality without requiring such large doses. Promisingly, recent work has provided encouragement that it is possible to measure apparent choroid plexus perfusion using ASL techniques in the human brain (Johnson et al., 2020; Zhao et al., 2020). Therefore, it may also be possible to apply these methods clinically, to investigate relative changes in CP perfusion in response to a drug or challenge, as performed here using the BCSFB-ASL approach.

It is important to consider the technical challenges to clinical translation of the method. As shown in our recent work (Evans et al., 2020) (Evans et al., 2020), the BCSFB signal across the lateral ventricles is markedly smaller than the standard ASL signal from the parenchymal tissue (primarily reflecting the relatively small volume of CP tissue within the lateral ventricles and the long TE). Nonetheless, we are able to detect relatively small signals from labelled blood water that has been delivered to the CSF compartment during the TI, in comparison to the signal detected from our TE = 20 ms (standard-ASL) measurement ΔM/M0 = 0.034 (±0.003) TE = 20 ms, vs 0.004 (±0.001) at TE = 220 ms (saline group average, *n* = 9). This corresponds to an estimated cortical perfusion of 243 (±8) ml/100 g/min and rate of BCSFB-mediated labelled blood water delivery to the CSF of 21.8 (±2.6) ml/100 ml/min.

In the human brain, reliable measurement of the BCSBF signal will be made challenging by the lower flow rates and increased arterial transit times relative to the mouse brain as well as the decreased T1 of the blood water at clinical field strengths. This will be offset, however, by the large increase in brain volume and CP tissue as well as modern sequence and hardware innovations such as multi-channel receiver coils. Encouragingly, measurements of the exchange time (~tissue transit time-arterial transit time) of labelled water across the BBB suggests similar timescales between the mouse and human (Gregori et al., 2013; Ohene et al., 2019), suggesting that the transfer of labelled water across the BCSBF should occur within clinically relevant ASL timing parameters (labelling duration and PLD) in the human brain.

An interesting feature of the measurement is that a low-resolution readout can be used to boost sensitivity due to lack of partial volume effects from blood and tissue at ultra-long TE. As such, the BCSBF-ASL technique aims to capture a measure of the total amount of BCSFB-mediated labelled blood water delivery to the lateral ventricles as a surrogate index of BCSFB function within the lateral ventricles. In this way the integral of the BCSFB signal is taken across the entire lateral ventricles meaning that this measure will be independent of ventricle volume and location of the CP within the lateral ventricles. Importantly, the low-resolution imaging comes at little cost since, unlike imaging of the blood brain barrier, where parenchymal vascular delivery often has high spatial affinity to the location of tissue metabolism, the measures of BCSFB function have little need for high spatial resolution because the material delivered from the blood to the CSF is immediately dispersed around the ventricular compartment due to CSF pulsation.

In conclusion, our results illustrate the value of an interleaved-TE ASL MRI approach to quantify pharmacologically-induced changes to vessels that make up the BBB in the cortex and the BCSFB in the choroid plexus. Caffeine appears to be a promising candidate to challenge the vasculature of the BCSFB, owing to the marked response of the BCSFB-ASL measurement to this safe and readily available drug. In response to vasopressin, an aged cohort displayed a marked impairment in BCSFB ‘reactivity’, relative to an adult cohort. Importantly, these results highlight the capability of such measurements to be utilised as a biomarker for probing altered functionality and pathophysiology in the aged or diseased brain, providing a potential novel biomarker of age-related cognitive decline.

## Data availability statement

Newly acquired imaging data for the present study is available through reasonable request to the lead or corresponding author.

## CRediT authorship contribution statement

**Charith Perera:** Conceptualization, Data curation, Investigation, Methodology, Formal analysis, Writing – original draft. **Ian F. Harrison:** Conceptualization, Writing – review & editing. **Mark F. Lythgoe:** Conceptualization, Resources, Writing – review & editing. **David L. Thomas:** Conceptualization, Supervision, Methodology, Writing – review & editing. **Jack A. Wells:** Conceptualization, Supervision, Funding acquisition, Investigation, Methodology, Writing – original draft, Project administration.

## Appendix Supplementary materials

Image, application 1

## References

[bib0001] Addicott M.A. (2009). The effect of daily caffeine use on cerebral blood flow: how much caffeine can we tolerate?. Hum. Brain Mapp..

[bib0002] Balusu S., Brkic M., Libert C., Vandenbroucke R.E. (2016). The choroid plexus-cerebrospinal fluid interface in Alzheimer's disease: more than just a barrier. Neural Regen Res.

[bib0003] Chen R.L. (2009). Age-related changes in choroid plexus and blood-cerebrospinal fluid barrier function in the sheep. Exp. Gerontol..

[bib0004] Dohrmann G.J. (1970). Dark and light epithelial cells in the choroid plexus of mammals. J. Ultrasructure Res..

[bib0005] Evans P.G. (2020). Non-Invasive MRI of Blood–Cerebrospinal Fluid Barrier Function. Nat. Commun..

[bib0006] Faraci F.M., Mayhan W.G., Farrell W.J., Heistad D.D. (1988). Humoral regulation of blood flow to choroid plexus: role of arginine vasopresin. Circ. Res..

[bib0007] Flueck J.L. (2016). Acute Effects of Caffeine on Heart Rate Variability, Blood Pressure and Tidal Volume in Paraplegic and Tetraplegic Compared to Able-Bodied Individuals: a Randomized. Blinded Trial.

[bib0008] Frolkis V.V., Kvitnitskaya-Ryzhova T.Y., Dubiley T.A. (2000). Vasopressin, hypothalamo-neurohypophyseal system and aging. Arch. Gerontol. Geriatr..

[bib0009] Gregori J., Schuff N., Kern R., Günther M. (2013). T2-based arterial spin labeling measurements of blood to tissue water transfer in human brain. J. Magn. Reson. Imaging.

[bib0010] Haller S. (2016). Arterial spin labeling perfusion of the brain: emerging clinical applications. Radiology.

[bib0011] Han M.-.E. (2009). Regulation of cerebrospinal fluid production by caffeine consumption. BMC Neurosci.

[bib0012] Hou Y. (2019). Ageing as a risk factor for neurodegenerative disease. Nature Reviews Neurology.

[bib0013] Hubert V. (2019). Clinical Imaging of Choroid Plexus in Health and in Brain Disorders: a Mini-Review. Front. Mol. Neurosci..

[bib0014] Hurford R. (2014). MRI-visible perivascular spaces: relationship to cognition and small vessel disease MRI markers in ischaemic stroke and TIA. J. Neurol. Neurosurg. Psychiatry.

[bib0015] Inoue Y., Tanaka Y., Hata H., Hara T. (2014). Arterial spin-labeling evaluation of cerebrovascular reactivity to acetazolamide in healthy subjects. Am. J. Neuroradiol..

[bib0016] Johanson C.E. (1999). AVP V1 receptor-mediated decrease in Cl- efflux and increase in dark cell number in choroid plexus epithelium. Am. J. Physiol. - Cell Physiol..

[bib0017] Johnson A.G., Crawford G.A., Kelly D., Nguyen T.V., Gyory A.Z. (1994). Arginine Vasopressin and Osmolality in the Elderly. J. Am. Geriatr. Soc..

[bib0018] Johnson S.E. (2020). Choroid plexus perfusion and intracranial cerebrospinal fluid changes after angiogenesis. J. Cereb. Blood Flow Metab..

[bib0019] Kastrup A., Dichgans J., Niemeier M., Schabet M. (1998). Changes of cerebrovascular CO2 reactivity during normal aging. Stroke.

[bib0020] Keck M.E. (2000). Ageing alters intrahypothalamic release patterns of vasopressin and oxytocin in rats. Eur. J. Neurosci..

[bib0021] Kety S.S., Schmidt C.F. (1948). The Effects of Altered Arterial Tensions of Carbon Dioxide and Oxygen on Cerebral Blood Flow and Cerebral Oxygen Consumption of Normal Young Men. J. Clin. Invest..

[bib0022] Kim S.-.G. (1995). Quantification of relative cerebral blood flow change by flow-sensitive alternating inversion recovery (FAIR) technique: application to functional mapping. Magn. Reson. Med..

[bib0023] Lein E.S. (2007). Genome-wide atlas of gene expression in the adult mouse brain. Nature.

[bib0024] Liszczak T.M., Foley L., Black P.M.L (1986). Arginine vasopressin causes morphological changes suggestive of fluid transport in rat choroid plexus epithelium. Cell Tissue Res.

[bib0025] Marques F., Sousa J.C., Sousa N., Palha J.A. (2013). Blood-brain-barriers in aging and in Alzheimer's disease. Mol Neurodegener.

[bib0026] Mitra J.K., Roy J., Sengupta S.Vasopressin (2011). Its current role in anesthetic practice. Indian Journal of Critical Care Medicine.

[bib0027] Nair A., Jacob S. (2016). A simple practice guide for dose conversion between animals and human. J. Basic Clin. Pharm..

[bib0028] Nakamura S., Hochwald G.M. (1983). Effects of arterial P(CO2) and cerebrospinal fluid volume flow rate changes on choroid plexus and cerebral blood flow in normal and experimental hydrocephalic cats. J. Cereb. Blood Flow Metab..

[bib0029] Nehlig A., de Vasconcelos A.P., Dumont I., Boyet S. (1990). Effects of caffeine, l-phenylisopropyladenosine and their combination on local cerebral blood flow in the rat. Eur. J. Pharmacol..

[bib0030] Ohene Y. (2019). Non-invasive MRI of brain clearance pathways using multiple echo time arterial spin labelling: an aquaporin-4 study. Neuroimage.

[bib0031] Page R.B., Funsch D.J., Brennan R.W., Herna ´ndez M.J. (1980). Choroid plexus blood flow in the sheep. Brain Res.

[bib0032] Prinzen F.W., Bassingthwaighte J.B. (2000). Blood flow distributions by microsphere deposition methods. Cardiovasc. Res..

[bib0033] Raichle M.E., Eichling J.O., Grubb R.L. (1974). Brain Permeability of Water. Arch. Neurol..

[bib0034] Raichle M.E., Grubb R.L. (1978). Regulation of brain water permeability by centrally-released vasopressin. Brain Res.

[bib0035] Redzic Z.B., Preston J.E., Duncan J.A., Chodobski A., Szmydynger-Chodobska J. (2005). The Choroid Plexus-Cerebrospinal Fluid System: from Development to Aging. Curr. Top. Dev. Biol..

[bib0036] Serot J.M., Foliguet B., Béné M.C., Faure G.C. (2001). Choroid plexus and ageing in rats: a morphometric and ultrastructural study. Eur. J. Neurosci..

[bib0037] Sturrock R.R. (1979). A morphological study of the development of the mouse choroid plexus. J. Anat..

[bib0038] Suri S. (2015). Reduced cerebrovascular reactivity in young adults carrying the APOE ε4 allele. Alzheimer's Dement.

[bib0039] van Dam R.M., Hu F.B., Willett W.C. (2020). Coffee, Caffeine, and Health. N. Engl. J. Med..

[bib0040] Van Der Flier W.M., Scheltens P. (2005). Epidemiology and risk factors of dementia. Neurology in Practice.

[bib0041] Vandenbroucke R.E. (2016). *Annals of the American Thoracic Society* vol. 13.

[bib0042] Vasostrict (2021). https://reference.medscape.com/drug/vasostrict-adh-vasopressin-342073.

[bib0043] Vercellino M. (2008). Involvement of the choroid plexus in multiple sclerosis autoimmune inflammation: a neuropathological study. J. Neuroimmunol..

[bib0044] Vidyasagar R., Greyling A., Draijer R., Corfield D.R., Parkes L.M. (2013). The effect of black tea and caffeine on regional cerebral blood flow measured with arterial spin labeling. J. Cereb. Blood Flow Metab..

[bib0045] Wagerle L.C., Mishra O.P. (1988). Mechanism of CO2 response in cerebral arteries of the newborn pig: role of phospholipase, cyclooxygenase, and lipoxygenase pathways. Circ. Res..

[bib0046] Williams J.L., Jones S.C., Page R.B., Bryan R.M. (1991). Vascular responses of choroid plexus during hypercapnia in rats. Am. J. Physiol. - Regul. Integr. Comp. Physiol..

[bib0047] Zhao L., Taso M., Dai W., Press D.Z., Alsop D.C. (2020). Non-invasive measurement of choroid plexus apparent blood flow with arterial spin labeling. Fluids Barriers CNS.

[bib0048] Zhou Y., Rodgers Z.B., Kuo A.H. (2015). Cerebrovascular reactivity measured with arterial spin labeling and blood oxygen level dependent techniques. Magn. Reson. Imaging.

