# Supplementary material for: Pharmacological MRI with Simultaneous Measurement of Cerebral Perfusion and Blood-Cerebrospinal Fluid Barrier Function using Interleaved Echo-Time Arterial Spin Labelling
Source: Neuroimage. 2021 Sep;238:118270. doi: 10.1016/j.neuroimage.2021.118270 (PMC8543042; doi:10.1016/j.neuroimage.2021.118270)
Supplement: Supplementary file 1 [file mmc1.docx]

**Supplementary Material**

**
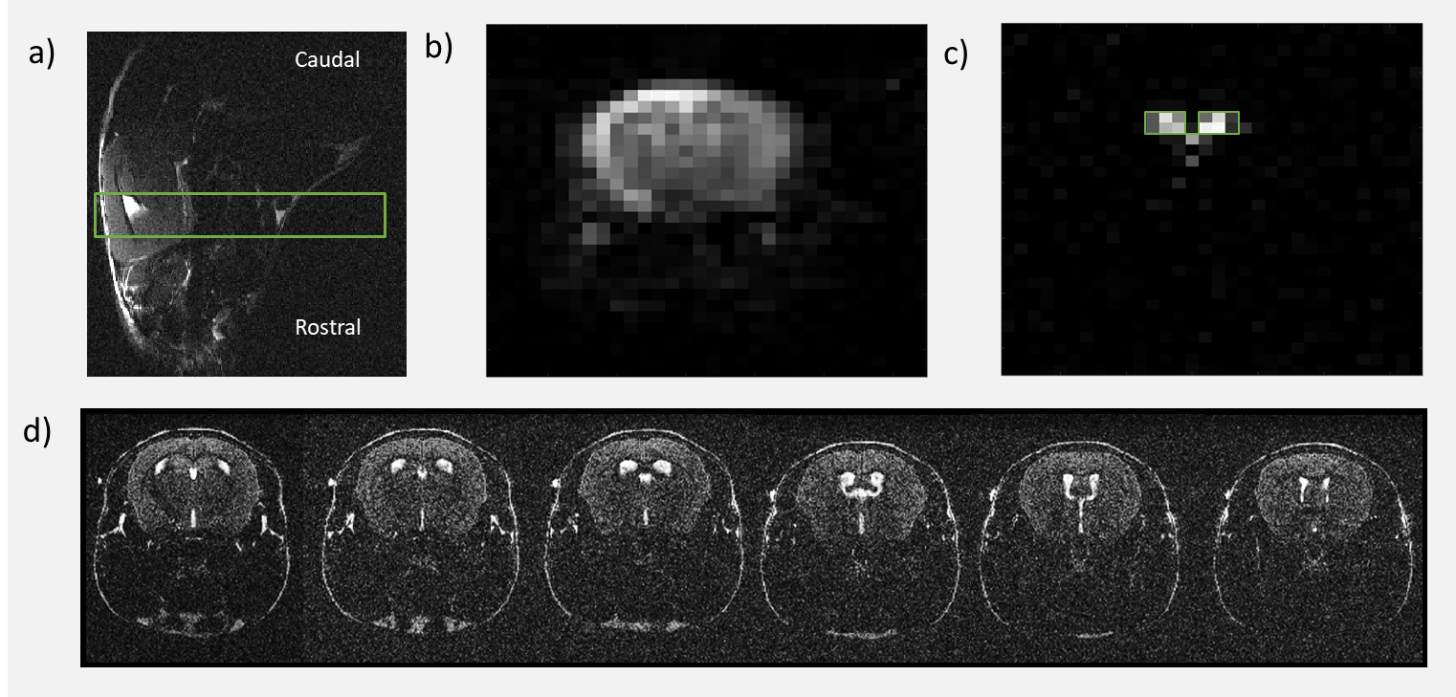
**

***Supplementary Figure 1 – Example MR images:*** *a) Sagittal anatomical reference image with overlaid FAIR-ASL imaging slice plan (single slice, 2.4mm). b) Subtracted (global – slice selective) interleaved-TE ASL image obtained at TE = 20 ms. c) Subtracted (global – slice selective) interleaved-TE ASL image obtained at TE = 220 ms with overlaid lateral ventricular ROI used during analysis. d) Coronal anatomical reference images displaying imaging volume across the ventricles (6x 0.4mm slices, 2.4mm total).*

**
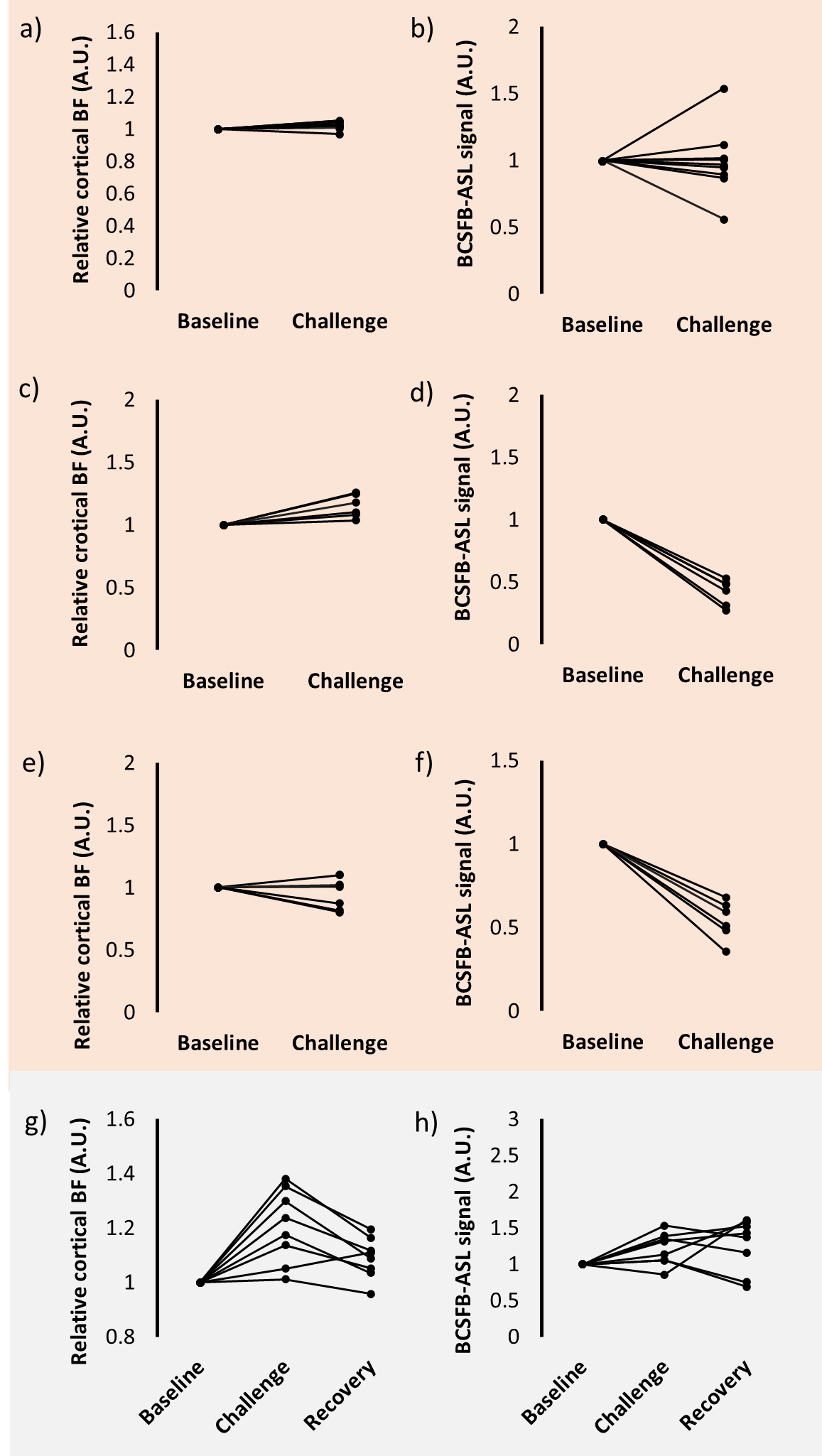
**

***Supplementary Figure 2 – Interleaved echo time ASL: cortical BF and BCSFB-ASL simultaneous responses to selected challenges.*** *Individual subject data showing baseline vs challenge comparison for relative cortical BF changes (left column: a, c, e, g) and BCSFB-ASL signal changes (right column: b, d, f, h). Top row: saline vehicles (n = 9), second row: vasopressin (n = 6), third row: caffeine (n = 6), bottom row: CO_2_ (n = 8).*

**
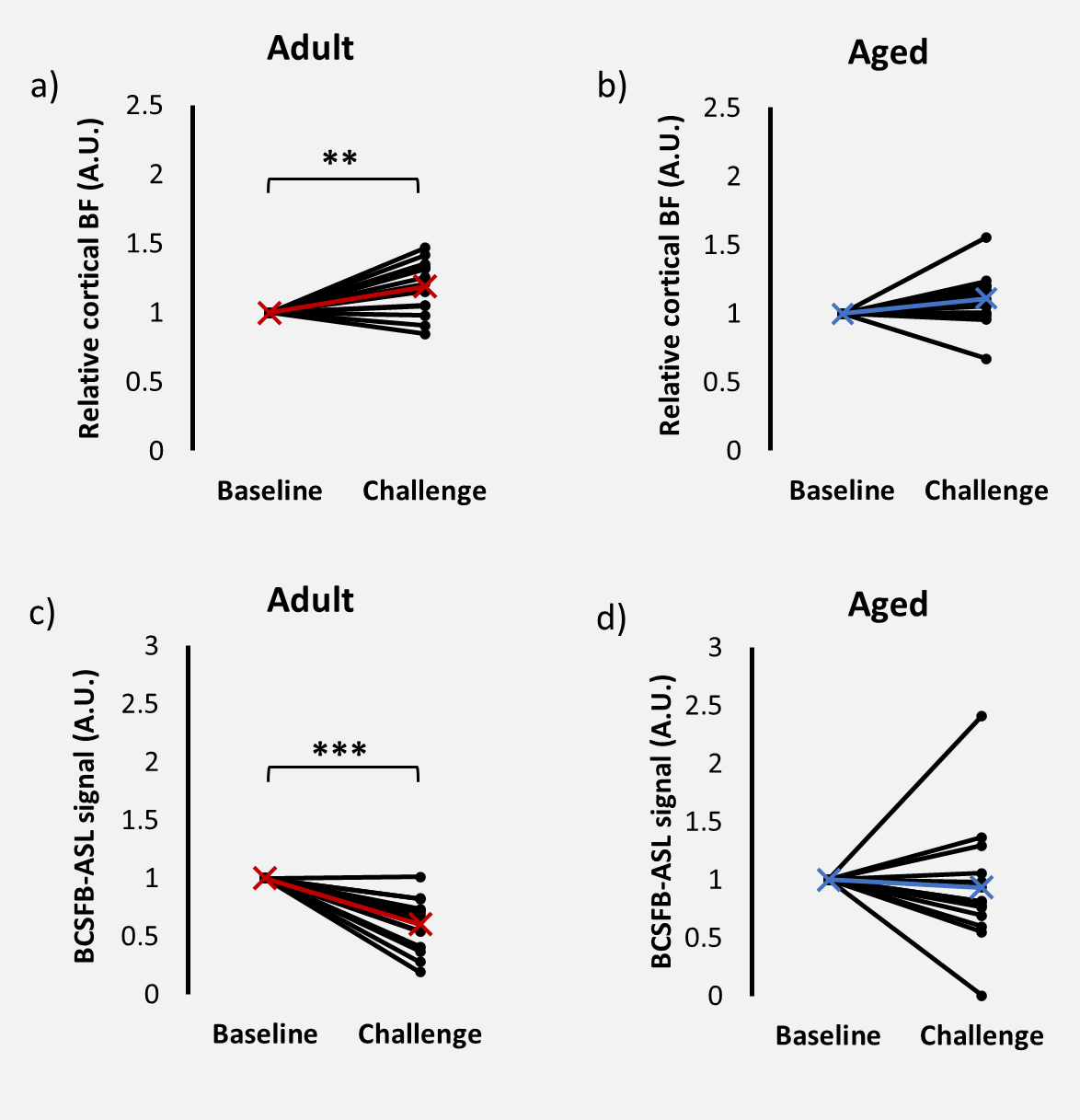
**

***Supplementary Figure 3 – Interleaved echo time ASL: adult vs aged response to vasopressin.*** *Ageing study individual subject data showing baseline vs challenge comparison for relative cortical BF changes (top row: a, b) and BCSFB-ASL signal changes (bottom row: c, d). Group-averaged baseline vs challenge values are shown for cortical BF (red line) and BCSFB-ASL signal (blue line).*
